# Wars and Suicides in Israel, 1948–2006

**DOI:** 10.3390/ijerph9051927

**Published:** 2012-05-18

**Authors:** Israel Oron (Ostre)

**Affiliations:** International Center for Health, Law and Ethics, Department of Psychology, Law and Ethics, University of Haifa, Mount Camel, Haifa 31905, Israel; Email: isr.oron@gmail.com

**Keywords:** Israel, suicide, existential and non-existential wars

## Abstract

This paper reports the characteristics of suicides which occurred during the existential and the non-existential wars in Israel. It provides a first approximation of whether the suicide patterns in each war are consistent with the findings of Morselli and Durkheim, and whether their theoretical interpretations can serve as a preliminary guideline to explaining the Israeli case, which is characterized by short periods of war, social integration during some of the non-existential wars, and a sharp rise in post-war male suicide rates following all of the existential wars. Implications for further studies on the subject in Israel and elsewhere are discussed.

## 1. Introduction

For nearly two centuries research in various academic fields has attempted to resolve the enigma of why people kill themselves by focusing on intra-psychic forces (e.g., psychopathology), as well as demographic and social parameters (e.g., age, gender, marital status, socio-economic level). These parameters have been shown to affect suicide, but in complex ways that are not easily understood. The effect of war, the utmost deadly man-made social event, on suicide, the utmost deadly man-made personal event, is a stimulating issue to study in the framework of suicidal behavior. 

### 1.1. Studies on Wars and Suicides: A Brief History

The pioneering work on this topic was conducted in the second half of the 19th century in a mental hospital by Enrico Morselli, an Italian physician. One of his goals was to examine the impact of social influences and psychological factors on suicide in Europe.

Morselli suggested there was a connection between a given political situation and personal reasons for committing suicide and analyzed statistical data on suicides during wars and revolutions. He found that during periods of turbulence the number of suicides decreased compared to the frequency of suicides in peace time. He inferred that the psychological cause which produced this effect was individuals’ preoccupation with their country’s fate during these periods. Morselli published his findings in 1879, in a book that was translated into English two years later [1].

Eighteen years later the French sociologist Emile Durkheim published his work on suicide [2]. He was also interested in the low frequency of suicides during wars and revolutions, and began his analysis with the statistical data and findings presented in Morselli’s studies. Durkheim confirmed these findings with additional data but pointed out that the drop in suicide rate was greater for more severe or prolonged crises, and *vice versa*. Durkheim proposed a social theory which relates suicide rates to the individual’s acceptance of societal goals. He postulated two mechanisms: social integration and social regulation. The first refers to the degree of acceptance of shared social convictions. The second refers to the degree of social control imposed by society on an individual's motives and feelings. He claimed that if a society operates well, uniformly and in a coordinated manner, it can monitor the behavior of individuals who contemplate ending their lives. War or profound social crises compel people to join forces to confront an external threat, and the individual focuses on the collective goal and not on himself/herself and his/her suicidal wish. This is in essence the social-integration model. 

It is important to note that Durkheim differentiated between an important or crucial war and one which does not elicit a broad consensus. A “Great National War” involves a territorial threat, arouses patriotic feelings and motivates the multitudes to come together to face the threat to their country. Only a war of this kind unites the people and thereby reduces suicide rates. The second type of war does not arouse collective emotions and there should not be a decrease in suicide rates. 

The twentieth century witnessed two world wars. The data show that suicide rates in France dropped during both wars [3], as was the case in Great Britain [4], and the United States [5]. The average drop in suicide rates for all nations involved in the First World War was 15.3% for the years 1915–1918, and in the Second World War, 13.5% for the years 1940–1945 (based on [6]).

Analysis of suicide rates during wars in the twentieth century also confirms Durkheim’s differentiation between types of war. For example, no reduction in suicide rates in the US population was found during the Vietnam War, which lasted from 1959 to 1975, and was waged by the Communist North against the South that was supported heavily by the US. The war did not endanger American territory or its population; hence the lack of decrease in suicide rates.

Certain other findings support the integration model [7,8,9,10,11,12,13,14,15,16], but a few have not [17,18,19,20].

### 1.2. Research Objectives

Regretfully, Israel has acquired the rather dubious reputation of a war-stricken country. During its entire existence it has been involved in no less than eleven wars. To date, no study has focused on the relationship of war and suicides in Israel, and the present one is the first inquiry into this topic [21,22,23]. Today, after six decades of wars and turbulent security situations, there continues to be a very solid foundation on which this issue can be examined.

The purpose of this paper is to portray the characteristics of completed suicides in the Hebrew/Jewish population in Israel as a whole which occurred during each of its existential and non-existential wars, and to reflect on the data against the relevant socio-political background for each.

Its second purpose is to provide a first approximation of whether the suicide patterns in each war are consistent with the findings of Morselli and Durkheim, and whether the social-integration interpretation (which still prevails in sociology) can serve as a preliminary and tentative guideline to explaining the Israeli case.

### 1.3. Israel’s Involvement in War

The wars are described and detailed below in chronological order, especially for those readers who are not familiar with the extensive history of Israel’s conflicts:

*War of Independence:* The official date of the outbreak of this war is the day the State was founded (14 May 1948), when the Arab armies invaded the new-born country to annihilate it. In fact, blood-letting incidents with Palestinian Arabs began as early as 29 November 1947, when they refused to accept and apply the United Nations resolution on the partition of Western Palestine into two states: Israeli and Arab. Hostilities actually ended in January 1949, but the cease-fire agreements were signed over the course of 1949. This war was the deadliest in the history of Israel, and claimed the lives of one percent of its population at the time (6,000 victims).

*Suez:* This war broke out as a result of the nationalization of the Suez Canal by Egyptian president Nasser. Until then navigation in the Canal was operated by a company, the majority of whose shares were owned by Britain. Britain and France planned to invade the Canal Zone in order to block nationalization, and informed Israel so that its penetration into the Sinai would serve as an excuse for the planned invasion. The Israeli operation began on 29 October 1956, and the battles ended on 5 November. In March 1957 the Israeli army evacuated the peninsula, and the British and French efforts also failed.

*The Six Day War:* In November 1966 a defense agreement was signed between Syria and Egypt, and in May 1967 the Syrians claimed that Israel was amassing military forces on its border. Although in fact this was untrue, an intelligence officer in the Soviet Embassy in Cairo confirmed the claim, disregarding the real state of affairs. As a result Egypt transferred military units to the Sinai Peninsula, and Nasser demanded the evacuation of UN Forces from the Sinai while threatening to annihilate Israel. Apprehension gripped the country’s population, reserve forces were called up, and a nerve racking military stand-by began until the outbreak of fighting on 5 June 1967.

*The War of Attrition:* Because of Egypt’s refusal to begin negotiations with Israel following the Six-Day War there was a period of political stagnation. At the same time it was clear to Egypt that its army was incapable of recapturing its territories now occupied by Israel. Against this backdrop Nasser decided to start a war limited to the Suez Canal line alone, a war which might prompt the US and the USSR to force a settlement upon Israel. Until that time, he hoped to be able to “deplete”, as he phrased it, Israel’s material and mental resources. Although since November 1968 a number of incidents had already taken place between the two countries, on 8 March 1969, artillery from both sides were used for the first time on the Canal front and on a broad scale. This incident marked the start of continuous military activity which lasted for 17 months, and at times included air force battles and commando raids by both parties. The fighting ended on 7 August 1970, with the acceptance of the American initiative for a cease-fire.

*The Yom Kippur War:* During the years following the end of the Six Day War it was claimed in Israel that Egypt was not capable of carrying out a military offensive or changing the status-quo between the two countries. Various political moves initiated by Egyptian president Sadat to settle the issue of the occupied Egyptian territories did not bear fruit, and he decided to launch a limited war in order to put the issue on the international agenda. Israeli Intelligence failed to interpret the signs of approaching war correctly and Israeli citizens were surprised by the outbreak of war on 6 October 1973.

*The First Lebanon War:* This war broke out on 5 June 1982, as a reaction to the attempt to murder the Israeli ambassador to Britain, and was conducted against the PLO which had established itself for some years in Lebanon. The war prompted a considerable anti-war movement in Israeli society which claimed that the attempted murder was not carried out by the PLO and that it was used solely as a pretext to attack its bases. This was the most disputed of all the wars of Israel, and during one anti-war demonstration a right-wing extremist murdered a demonstrator and injured a few others. Formally, the hostilities, which were confined to Lebanese territory, ended in August 1982 with the PLO’s exit from Lebanon, but in fact the fighting continued sporadically and resulted in hundreds of victims. About two years later the Israeli Defense Force positioned itself in a security strip in Lebanon, and only withdrew to the international border in May 2000.

*The First Intifada:* (the word connotes an uprising). In 1982, in the framework of the Peace Settlement, Israel gave back most of the territories occupied during the Six-Day War to Egypt. One dispute remained—where to place the international border in Taba—and towards the end of 1986 both governments negotiated to solve the dispute. Eventually the Egyptian demand prevailed. At the same time, “autonomy talks” with Egypt, whose purpose was to create a new political situation in the West-Bank and Gaza as an interim stage to determine their final status, ran aground. However, the Israeli government did not have the political clout to make decisions on the Palestinian issue due to the standoff between the equal numbers of Left and Right parties in parliament.

Thus, from the point of view of Palestinian society, it did not follow in the footsteps of the (Egyptian) precedent of territories in exchange for political settlement, as the political establishment in Israel did little to resolve the state of affairs. This set the stage for violent protests by Palestinians which began in December 1987 against IDF soldiers in the West-Bank and Gaza, and especially against settlers in these areas as well as civilians within the international borders of the country. The Gulf War which broke out in mid-January 1991 put an end to these acts.

*The First Gulf War:* This war differed from previous ones by the fact that the threat to the country was located hundreds of kilometers away, following the invasion of USA forces in Iraq after the latter conquered Kuwait. Furthermore the IDF did not take part. 

*El-Aksa Intifada:* This second Palestinian uprising began in 29 September 2000, following the failure of the Camp David talks (July 2000) under the auspices of President Clinton, and one day after opposition leader Ariel Sharon visited the Temple Mount in Jerusalem. In January 2005 Mahmud Abbas (the elected Chairman of the Palestinian Authority after Arafat’s death in November 2004) met with PM Sharon (elected in March 2001). Both sides announced an end to the violence.

*The Second Gulf War:* This war was spearheaded by the US to eliminate weapons of mass destruction in Iraq and end Saddam Hussein’s support of terrorism, as President Bush put it. The war lasted from 20 March to 1 May 2003, and Israel abstained from any military action.

*The Second Lebanon War:* The military conflict began on 12 July the 2006, when Hezbollah killed three Israeli soldiers on its northern border with Lebanon, and two others were wounded, captured, and taken to Lebanon. A cease fire went in effect on 14 August 2006.

## 2. Method

The eleven wars can be classified into three groups. Group One includes existential wars which were conducted with a clear apprehension of an imminent military defeat and the ensuing annihilation of the State: the War of Independence, the Six-Day War and the Yom-Kippur War. The remaining wars do not fit the category of total war. Group Two covers wars (the Suez war, the War of Attrition, and the Lebanon Wars) that were conducted outside Israel or on its borders. Group Three covers the first and the second Intifada and the two Gulf Wars, which were characterized by an absence of battles between armies and deliberate attacks on civilians (not included in this paper). 

Information regarding suicide data in Israel since 1948 onward was collected from the Ministry of Health and from the Central Bureau of Statistics. Some of the data were processed and summarized by the author (figures were calculated here only for the Hebrew/Jewish population due to the very low suicide rate in the Arab minority in Israel. To have used a combined rate in this connection would have meant assigning a rate to a population in the majority which is artificially much lower than the true state of affairs).

## 3. Results

The Table 1 and Table 2 present the suicide rates for the first two groups of wars, showing the incidence of suicides per 100,000 Hebrew inhabitants aged 15 and above. Each of them compares (in percentages) the national suicide rate and that of both genders (*i.e.*, per 100,000 for each gender) for every year in which a war occurred, with the figures for the previous (left-hand side) and subsequent years (right-hand side). Suicides of soldiers are included. 

## 4. Discussion

### 4.1. Existential Wars

*War of Independence:* Suicide rates declined during this war (since the suicide data for 1947 are missing I therefore used the rates calculated for the year 1946, which are a close estimate to those that might have been found for 1947 [24,25]). The rise in suicide rates for men in 1949 may be explained by Durkheim’s interpretation that after a crisis is over, the number of suicides rises. Taking into consideration the mass immigration into Israel during 1949, which increased the population by 50%, the estimated increase in suicide rates for males living in the country during the war is about 45%. For this reason, the post-war decrease in female suicide rates is assumed to nearly cancel out.

**Table 1:** Suicides during existential wars.

Wars	Duration	Prewar Year				Postwar			
			National	Male	Female	Year	National	Male	Female
War of	8 months	(1946)	(14.9)	(15.1)	(14.6)	**1948**	14.0	14.3	14.0
Independence	(5/14/1948–1/7/1949)	**1948**	14.0	14.3	14.0	1949	17.7	24.0	10.9
Change %			–6.0%	–5.3%	–4.1%		26.4%	67.8%	–22.1%
Six Day War	6 days	1966	9.9	11.9	7.9	**1967**	9.9	12.8	7.0
	(6/5/1967–6/10/1967)	**1967**	9.9	12.8	7.0	1968	14.1	15.9	12.4
Change %			0.0%	7.60%	–11.4%		42.4%	24.20%	77.1%
Yom Kippur War	37 days	1972	13.0	13.9	12.0	**1973**	9.4	10.9	7.9
	(10/6/1973–11/11/1973)	**1973**	9.4	10.9	7.9	1974	11.5	14.5	8.5
Change %			–27.7%	–21.6%	–34.2%		22.3%	33.0%	7.6%

**Table 2:** Suicides during non-existential wars.

Wars	Duration	Prewar				Postwar			
		Year	National	Male	Female	Year	National	Male	Female
Suez War	10 days	1955	15.3	13.3	17.2	**1956**	20.4	23.8	16.9
	(10/29/1956–11/5/1956)	**1956**	20.4	23.8	16.9	1957	14.4	16.7	12.0
Change %			33.3%	78.9%	–1.7%		–29.4%	–29.8%	–28.9%
War of	17 months	1968	14.1	15.9	12.4	**1970**	10.9	12.4	9.4
Attrition		**1969**	13.8	16.5	11.0	1971	12.5	13.4	11.5
Change %	(3/8/1969–8/7/1970)		–2.1%	3.8%	–11.3%		14.7%	8.1%	22.3%
		**1969**	13.8	16.5	11.0				
		**1970**	10.9	12.4	9.4				
Change %			–21.0%	–24.8%	–14.6%				
1st Lebanon War	2 months	1981	8.8	11.3	6.4	**1982**	8.5	10.1	6.2
	(6/5/1982–8/18/1982)	**1982**	8.5	10.1	6.2	1983	10.2	13.8	6.7
Change %			–3.4%	–10.6%	–3.1%		20.0%	36.6%	8.1%
2nd Lebanon	34 days	2005	9.7	14.2	5.5				
War	(7/12/2006–8/14/2006)	**2006**	8.2	12.7	4.0	No	data are	available	yet
Change %			–15.5%	–10.6%	–27.3%				

From a methodological point of view, testing the statistical significance of the difference between pre-war and post-war suicide rates is not relevant to data that cover a complete population.

**Figure 1:**
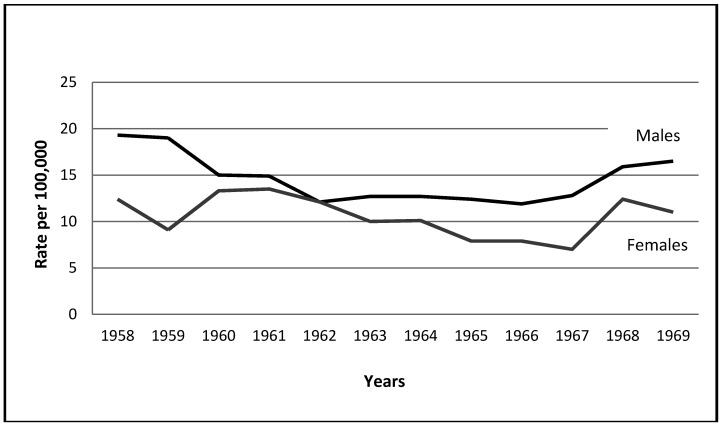
Time-plot: The Six-Day War (6/5/1967–6/10/1967).

*The Six-Day War:* The increase in the male suicide rate in 1967 by 7.6% should be compared to the variation in rates from 1958 (following the immediate impact of the Suez War in 1956–1957) onward up to 1966. This needs to be done in order to be positively conclusive that the observed increase is a result of the impact of war and not of a trend, or fluctuations due to events which took place during the intervening years between these two wars. Figure 1, which plots these rates for the relevant years, shows clearly that from 1958 on, there was a distinct decrease in suicide rates which only ended in 1967 in increased suicide rates.

A slight increase during this period of peace can be seen between the years 1962 and 1963, but it does not disconfirm the general pattern especially since it is less than 2.6%, compared to the increase in 1967. 

The claim that the increase in male suicides in 1967 was due to the impact of the war is supported by the finding that rates continued to increase following the war by 24.2%, and still further in 1969, by 3.8%. 

The decrease in female suicide rates by 11.4% in 1967 does not seem to be related to the war, because in four out of nine years after 1958 there were similar decreases. In fact, two decreases are much more striking: 26.6% between the years 1958–1959, (to be followed by a 46.2% increase), and 21.8% between 1964–1965.

As is clear in Figure 1, the impact of the war also affected female rates following the war, as shown by a 77.1% increase. This is the highest increase for female rates in Israel’s entire existence. This post-war increase could arguably be rooted in the conditions of war which induce severe stress that can affect the personality of soldiers and civilians alike. As the data show, the strain of day-to-day routines, which usually correlates with suicide, does not impact to such a degree on suicide rates.

From Durkheim’s point of view the findings for both genders in this war are contrary to the existential threat felt by the entire population during May 1967. Even if this may stem from the short duration of the fighting and the swift victory which immediately ended the existential threat from July onward, the overall pattern runs counter Durkheim’s hypothesis.

**Figure 2:**
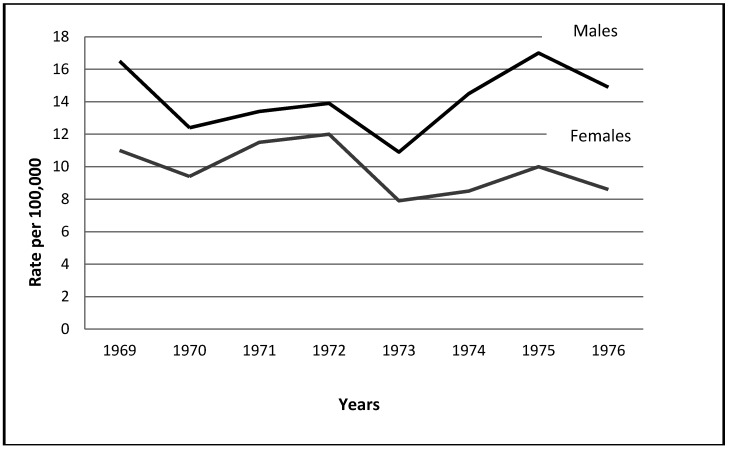
Time-Plot: The Yom–Kippur war (included: The War of Attrition) (10/6/1973–11/11/1973), (3/8/1969–8/7/1970).

*The Yom Kippur War:* Compared to the rates in the peaceful years which followed the Six Day War and the War of Attrition, there was a substantial decrease in male as well as female suicide rates in 1973, as shown by the figures pertaining to the war. The findings are consistent with the integration model. A time plot for this war, which includes the War of Attrition as well, is depicted in Figure 2. 

The marked post-war increase in male suicides highlights an essential difference with existential wars examined around the world. Whereas the common pattern of post-war increases in other wars normally does not exceed 15% (including wars examined by Morselli and Durkheim), in Israel all the male rates following all of the existential wars, and female rates after the Six Day War, were far above this level.

Figure 2 shows the magnitude of the increase which continued in 1975, and added about 17% to the post war rates of both genders. Thus the “great national wars” had a specific impact on Israeli society: post war rates not only returned to their prewar point of departure, but the turbulent atmosphere of the existential wars prompted more people to carry out their suicidal plans. 

### 4.2. Wars over the Border

*Suez:* Male suicide rates increased in 1956. The decrease in female rates was not limited to that year of the war, as three more decreases in female rates occurred after 1949. The findings for both genders are in line with Durkheim’s interpretation of suicide rates in a non-crucial war. 

Nevertheless, it should be noted that 1955 was a unique and never-repeated year in Israel’s existence, in which female suicides exceeded those of males. This fact could complicate data analysis as compared to other wars, given that there was an unusual peak in rates which then returned to its “natural” baseline during the next few years.

*The War of Attrition:* A negative difference was found in women’s suicide rates in 1969, and an overall decrease in rates in 1970 (see Figure 2). Contrary to Durkheim’s view, the decrease, coupled with increases in rates in 1971, point to the conclusion that even though this war was waged far away from the international border with Egypt, it triggered collective cohesiveness due to the socio-political atmosphere of that time. This is explained first of all by the dual fact that the War of Attrition contrasted sharply with the swift victory in the Six-Day War and it was the first time that Israel had faced a prolonged period of continuous low intensity hostilities. In addition, the population was bewildered by a situation that could not be won by either military or political means, while pointless exchanges of fire went on and on. Although daily life continued, the psychological stress increased, fueled in particular by reports of dead and wounded reserve and regular soldiers. This may have set the stage for feelings of solidarity and thereby a higher level of social cohesion, which in turn prevented suicides. In other words, such a continuous and insoluble war was gradually perceived as a nerve-racking period of time which threatened the wellbeing of society and elicited solidarity, even though the existence of the country as a whole was not in danger.

*The 1st Lebanon War:* Unlike the preceding non-existential wars, this was the first conflict which was waged unanimously. How did this fact impact suicide rates?

**Figure 3:**
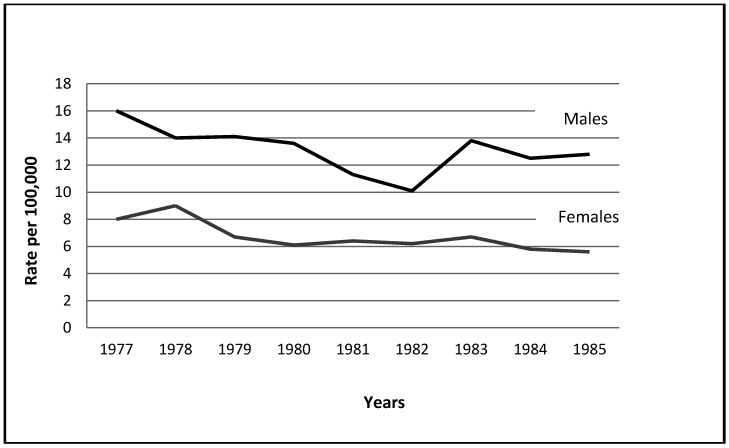
Time-Plot: The 1st Lebanon War (6/5/1982–8/18/1982).

Figure 3 illustrates conclusively an unprecedented increase in male suicides following the war, compared to the obvious decrease from 1977 to 1982. Aside from the three existential wars no other war in Israel had the same effect. This controversial and hotly disputed war may have taxed men heavily, much as did the existential wars.

The magnitude of decrease for both genders in 1982 resembles other decreases during the period since 1977. The findings fit Durkheim's interpretation of non-existential war that does not generate patriotic enthusiasm. 

*The 2nd Lebanon War:* Suicide rates for both genders decreased in this war compared to 2005, the sole conflict-free year following the second Intifada.

The war was initiated by the Israeli government with the intention to conduct it exclusively on Lebanese soil, as was the first one, but it soon escaped the government’s control. Hezbollah in southern Lebanon managed to strike back by firing barrages of missiles at the north of Israel, and threatened to strike the city of Tel-Aviv and its populated environs in the center of Israel. Consequently, everyone in those areas was gripped by a personal existential threat which could explain the lower rates of suicides as a result of social integration.

## 5. Conclusions

Ever since wars have had such a wide impact on human behavior, it is no surprise that war should also influence suicide. The findings presented here address the issue of wars and suicides in Israel, and highlight a number of characteristics specifically pertaining to the Israeli situation.

First, researchers around the world have compared suicide rates in peace time, which is defined as an absence of all hostilities. However, in Israel there has never been complete peace. In all its years of existence there has never been a single year without incidents, and in each of its years, without exception, soldiers or civilians have been killed or injured in various hostilities. 

Second, more than one war has broken out in Israel simultaneously with the end of the previous one. The War of Independence, for example, broke out without a halt in the sequence of bloody incidents that began in 29 November 1947, while the general population and, in particular, the Holocaust survivors who had immigrated to the country were still coping with the effects of World War II. Similarly, the outbreak of the Gulf War put a halt to the first Intifada.

In addition, many wars conducted by Israel lasted several days or weeks. The impact of wars of this type on the extent of social integration and the level of suicide rates in comparison to lengthy wars has not been studied. The Six-Day War is a good example.

The data and the interpretations in this study can serve as a basis for specific insights and hypotheses in subsequent studies, which should not be limited to the original social integration model or its variants.

In particular, in order to understand the mental state of suicidal individuals during wars (in Israel and elsewhere), psychological studies are of great importance, since sociological models alone fail to achieve this purpose. These two different but complementary angles could contribute to a meaningful understanding of demographic data, and might unravel different kinds of motivation and threats besides social integration that result in decreases in suicide rates in wars. By the same token, the focus of attention should also be on those who commit suicide in times of wars and national solidarity. 

In either method of inquiry attention should be paid to the differential contribution of gender and age groups to the rates, as well as to the Arab minority that shares in the ethnicity of the wider Arab region and presumably was influenced differently from the Hebrew population by periods of armed conflict. Undoubtedly, much remains to be done to unravel the complicated routes by which wars affect suicides. 
